# E-vita OPEN NEO in the treatment of acute or chronic aortic pathologies: first interim results of the NEOS study

**DOI:** 10.1093/ejcts/ezae206

**Published:** 2024-06-03

**Authors:** Konstantinos Tsagakis, Joerg Kempfert, Andreas Zierer, Andreas Martens, Daniel-Sebastian Dohle, Alessandro Castiglioni, Randolph Hung-Leung Wong, Kazimierz Widenka, Oliver Liakopoulos, Michael A Borger, Aung Ye Oo, Tomas Holubec, Maximilian Luehr, Juan José Legarra Calderón, Martin Grabenwöger

**Affiliations:** Department of Thoracic and Cardiovascular Surgery, West-German Heart and Vascular Center, University Hospital Essen, Essen, Germany; Department of Cardiothoracic and Vascular Surgery, Deutsches Herzzentrum der Charité (DHZC), Berlin, Germany; Charité—Universitätsmedizin Berlin, corporate member of Freie Universität Berlin and Humboldt-Universität zu Berlin, Berlin, Germany; Department of Cardiovascular and Thoracic Surgery, Faculty of Medicine, Johannes Kepler University Linz, Linz, Austria; Department of Cardiovascular and Thoracic Surgery, Wels-Grieskirchen Clinic, Wels, Austria; Clinic of Cardiac Surgery, University Hospital Oldenburg, Oldenburg, Germany; Department of Heart and Vascular Surgery, University Medical Center of the Johannes Gutenberg-University Mainz, Mainz, Germany; Department of Cardiac Surgery, IRCCS San Raffaele Hospital, Vita-Salute San Raffaele University, Milan, Italy; Division of Cardiothoracic Surgery, Department of Cardiothoracic Surgery, the Chinese University Hong Kong, Hong Kong; Department of Cardiac Surgery, District Hospital No. 2, University of Rzeszów, Rzeszów, Poland; Departemnt of Cardiac Surgery, Kerckhoff-Clinic, Campus Kerckhoff, University of Gießen, Bad Nauheim, Germany; University Department of Cardiac Surgery, Heart Center Leipzig, Leipzig, Germany; St Bartholomew’s Hospital, Barts Health NHS Trust, London, UK; Department of Cardiovascular Surgery, University Hospital Frankfurt, Goethe University, Frankfurt, Germany; Department of Cardiothoracic Surgery, Heart Center of the University of Cologne, Cologne, Germany; Department of Cardiovascular Surgery, University Hospital Álvaro Cunqueiro, Vigo, Spain; Department of Cardiovascular Surgery, Clinic Floridsdorf, Vienna, Austria; Institute of Cardiovascular Research, Karl Landsteiner Society, Vienna, Austria; Medical Faculty, Sigmund Freud Private University, Vienna, Austria

**Keywords:** Frozen elephant trunk procedure, Aortic arch aneurysm, Aortic dissection, E-vita OPEN NEO, Antegrade cerebral perfusion, Distal aortic perfusion

## Abstract

**OBJECTIVES:**

The aim of this multicentre study was to demonstrate the safety and clinical performance of E-vita OPEN NEO Stent Graft System (Artivion, Inc.) in the treatment of aneurysm or dissection, both acute and chronic, in the ascending aorta, aortic arch and descending thoracic aorta.

**METHODS:**

In this observational study of 12 centres performed in Europe and in Asia patients were enrolled between December 2020 and March 2022. All patients underwent frozen elephant trunk using E-vita OPEN NEO Stent Graft System. Primary end point was the rate of all-cause mortality at 30 days and secondary end points included further clinical and safety data are reported up to 3–6 months postoperatively.

**RESULTS:**

A total of 100 patients (66.7% male; mean age, 57.7 years) were enrolled at 12 sites. A total of 99 patients underwent surgery using the E-vita OPEN NEO for acute or subacute type A aortic dissection (*n* = 37), chronic type A aortic dissection (*n* = 33) or thoracic aortic aneurysm (*n* = 29), while 1 patient did not undergo surgery. Device technical success at 24 h was achieved in 97.0%. At discharge, new disabling stroke occurred in 4.4%, while new paraplegia and new paraparesis was reported in 2.2% and 2.2%, respectively. Renal failure requiring permanent (>90 days) dialysis or hemofiltration at discharge was observed in 3.3% of patients. Between discharge and the 3–6 months visit, no patients experienced new disabling stroke, new paraplegia or new paraparesis. The 30-day mortality was 5.1% and the estimated 6-month survival rate was 91.6% (standard deviation: 2.9).

**CONCLUSIONS:**

Total arch replacement with the E-vita OPEN NEO can be performed with excellent results in both the acute and chronic setting. This indicates that E-vita OPEN NEO can be used safely, including in the setting of acute type A aortic dissection.

## Graphical Abstract

**Figure:**
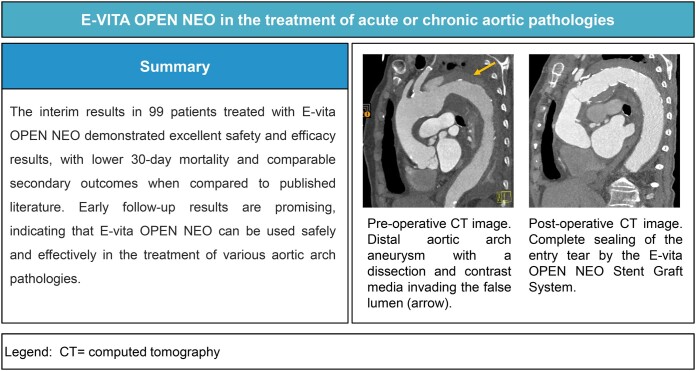

## INTRODUCTION

Treatment options for patients with thoracic aortic pathology involving the aortic arch has broadened substantially over the last decades. Traditionally, the domain of treatment for aortic arch pathology was open cardiac surgery [1]. Despite significant advances in medical therapy, repair of aortic arch disease using conventional treatment remains a surgical challenge and is still associated with significant incidence of perioperative mortality and neurologic complications. Furthermore, many patients are denied surgical interventions owing to significant morbidities and high surgical risk [2].

The introduction of the frozen elephant trunk (FET) technique since 1996 has changed the landscape of the treatment of the complex aortic arch disease [3]. It is a modification of the conventional elephant trunk technique and enables a single-stage repair of aortic pathologies. The FET technique is a hybrid procedure that allows the simultaneous repair of extensive segments of the aorta using a prothesis consisting of a proximal polyester segment attached to a distal stent graft. It is applicable in acute and chronic aortic dissections to stabilize the true lumen and cover the entry/re-entry sites along the proximal half of the descending aorta [4].

In 2005, the E-vita OPEN PLUS stent graft hybrid prothesis (JOTEC GmbH, Hechingen, Germany, a wholly owned subsidiary of Artivion, Inc., Kennesaw, USA) became the first commercially available FET hybrid prothesis. This device was later succeeded by E-vita OPEN NEO Stent Graft System (JOTEC GmbH, Hechingen, Germany, a wholly owned subsidiary of Artivion, Inc., Kennesaw, USA) which received CE approval in 2020. Similar to E-vita OPEN PLUS, the E-vita Open NEO graft consists of 2 vascular prosthetic components as a continuous tube: a proximal surgical prosthesis for the arch and a distal self-expandable stent graft with Z-nitinol stents for the descending aorta. The E-vita Open Neo does not contain any coating or any metal knitting. The graft is delivered by a flexible atraumatic introducer through the open aortic arch into the lumen of the descending aorta. The delivery system consists of a control handle, an atraumatic tip, a flap with protective wire, a graft protector, a pusher, a release handle, a release trigger and a release wire. The E-vita OPEN NEO is a hybrid aortic arch device for the repair and replacement of the thoracic aorta using the FET procedure which provides an alternative for conventional open surgery. The E-vita OPEN NEO is available in a range of sizes with varying diameters and lengths and comes in 3 different configurations for individual supra-aortic vessel management. Indications for the FET procedure include acute and chronic aortic dissections as well as aortic aneurysm with involvement of the arch and/or proximal descending aorta.

The NEOS study prospectively evaluates the safety and efficacy of E-vita OPEN NEO Stent Graft System in the treatment of aneurysm or dissection in the ascending aorta, aortic arch and descending thoracic aorta. Patient data are documented at preoperative planning, intervention, discharge from hospital, 3–6 months and 12-, 24-, 36- and 60-month follow-up. This manuscript describes the clinical and safety data of 100 enrolled patients collected until 3- to 6-month visit.

## METHODS

### Study design and patient population

The NEOS study is an observational, prospective, non-randomized, multicentre study, evaluating the safety and efficacy of the E-vita OPEN NEO Stent Graft System. Inclusion and exclusion criteria of the protocol are given in https://clinicaltrials.gov/study/NCT04676672. Between December 2020 and March 2022, patients with aneurysm or dissections in the ascending aorta, aortic arch and descending thoracic aorta were screened in 11 centres in Europe and in 1 centre in Hong Kong. Enrolment took place after the physician had made the decision to treat the patient with E-vita OPEN NEO Stent Graft System and the patient had agreed to undergo the intervention.

A contrast-enhanced spiral computed tomography scan is performed for preoperative planning, after the intervention at discharge, at 3–6 months, at 12 months (±2 months), at 24 months (±2 months), 36 months (±2 months) and 60 months (±2 months) follow-up. Data from blood and clinical examination are collected during follow-up visit on time points mentioned above. computed tomography scans and final angiograms were analysed by an independent CoreLab (Syctactx, LLC, New York, USA).

### Study end points and definition

The objective of the NEOS study is to evaluate the safety and clinical performance of E-vita OPEN NEO in the treatment of aneurysm or dissection in the ascending aorta, aortic arch and descending thoracic aorta.

Primary and secondary end points were defined in the study protocol (https://clinicaltrials.gov/study/NCT04676672). The primary safety end point is the rate of all-cause mortality at 30 days after the index procedure. Safety and feasibility at after intervention to discharge visit and at 3- to 6-month follow-up were evaluated based on clinical end points including device technical and treatment success, mortality, new disabling stroke, new paraplegia, new paraparesis and renal failure requiring permanent (>90 days) dialysis or hemofiltration.

Device technical success related to E-vita OPEN NEO is assessed 24 h after the index procedure (intubation) and combines the following criteria: successful delivery (i.e. ability to deliver the implant to the intended location without the need for unanticipated corrective intervention related to delivery); successful and accurate deployment of the device, as defined: deployment of the device in the planned location without the need for unanticipated corrective intervention related to deployment, patency of the device, absence of device deformations (e.g. kinks, collapse, stent eversion, mal-deployment, mis-aligned deployment), absence of device failure (e.g. fracture, fabric wear, graft hole or tear) requiring unplanned interventionand successful withdrawal (i.e. successful withdrawal of the delivery system, without the need for unanticipated corrective intervention related to withdrawal).

Treatment success is assessed at discharge and all follow-up visits and combines the following criteria: device technical success without: aortic-related mortality, aortic rupture, aortic enlargement in the region of the primary treated lesion, stent graft-induced new dissection, fistula formation, loss of device integrity; the following subset of major adverse events which include new disabling stroke within 3–6 months of procedure (modified Rankin Scale (mRS) ≥2 at 3–6 months follow-up and mRS <2 at baseline); new paraplegia (modified Tarlov Scale ≤2 at follow-up and >2 at baseline); and new paraparesis (modified Tarlov Scale 3 or 4 at follow-up and 5 at baseline).

New disabling stroke, new paraplegia and new paraparesis are evaluated at 2 different timepoints: after intervention to discharge visit and after discharge to 3–6 months visit. Renal failure requiring permanent (> 90 days) dialysis or hemofiltration is assessed only at discharge visit. New disabling stroke is defined as mRS ≥2 at follow-up and mRS <2 at baseline. New paraplegia (>30 days) is further explained as modified Tarlov Scale ≤2, and new paraparesis (>30 days) is identified as modified Tarlov Scale 3 or 4.

The EuroSCORE II was referred to define the urgency of the surgery, where elective is defined as routine admission for operation; urgent as patient who required intervention or surgery on the current admission and cannot be sent home; and emergent as operation before the beginning of the next working day.

### Operative strategy

**Figure 1:**
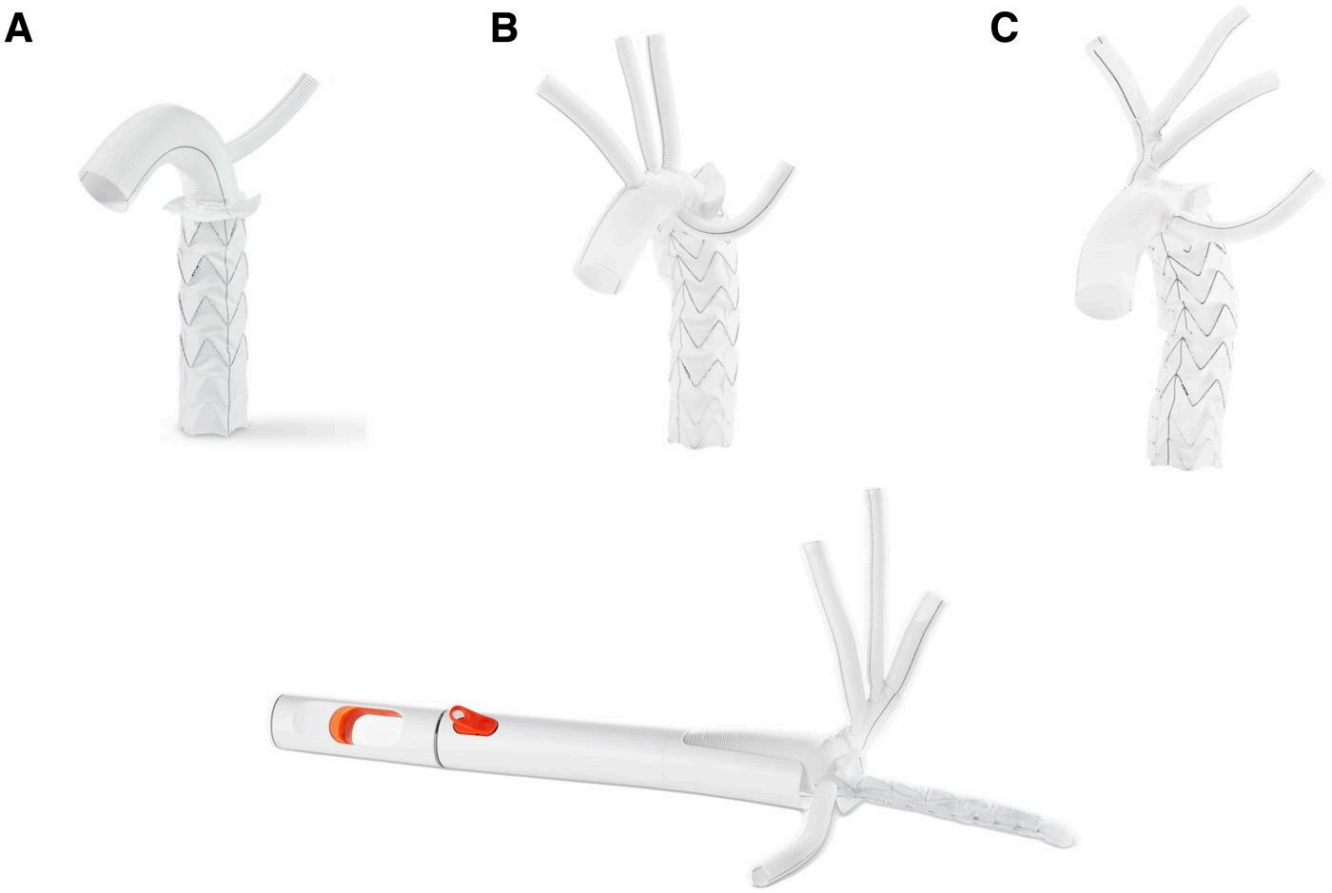
E-vita OPEN NEO Stent Graft System. (**A**) Images of the E-vita OPEN NEO Stent Graft System in three configurations (branched, straight, trifurcated) and the delivery system.

**Figure 2:**
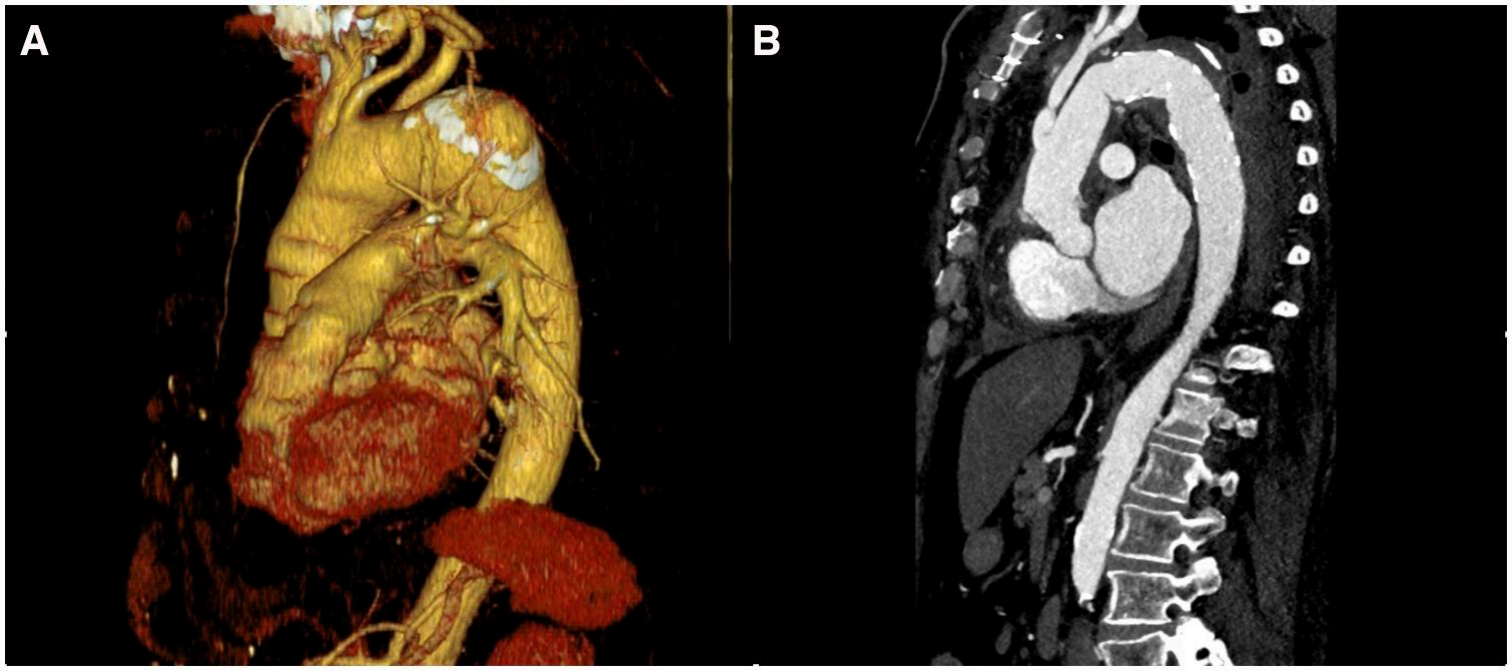
(**A**) Preoperative CT image. 3D reconstruction of an atherosclerotic aneurysm of the distal arch. (**B**) Postoperative CT image. Complete exclusion of the atherosclerotic aneurysm by the frozen elephant trunk procedure. CT: computed tomography.

Surgeons followed the standard of care during the FET surgery. All operations were performed under general anaesthesia and through median sternotomy. There was no standardized protocol for the surgery between the centres. There are 3 different configuration of the E-vita OPEN NEO Stent Graft System used in this study (Fig. 1). With the application of the E-vita OPEN NEO Stent Graft System, we aimed to exclude aneurysm and dissection in the patients (Fig. 2A and B).

### Statistical analysis

The interim analysis was done on locked databases (soft lock), with all queries relative to the safety data resolved at the time of the given export of data for all subjects and data available. This resulted in data being available for the first 100 enrolled patients who reached 3–6 months follow-up visits at the time of data transfer. Primary analysis set for statistical reporting was the modified intent-to-treat population with no replacement of missing data planned in the statistical analysis to provide unbiased results. Continuous variables were summarized using standard quantitative statistics: number of missing and non-missing observations, mean, standard deviation, median, quartiles and range (minimum and maximum observed values). Categorical variables were summarized using classical frequency statistics: number of missing and non-missing observations and percentages by categories. Percentages will be calculated on the number of non-missing observations. When applicable, bilateral asymptotic or exact confidence intervals for binomial distributions were calculated at the 95% level (unadjusted 95% confidence interval). Estimated calculation of survival was performed by the Kaplan–Meier analysis. The Kaplan–Meier analysis does not consider competing risk. Thus, this approach taken has a limitation and the estimated survival rate might be imprecise. Statistical analyses were performed using SAS version 9.4 (SAS Institute Inc.).

### Ethics

The NEOS registry was conducted according to the Declaration of Helsinki. Where required, the study protocol and patient informed consent form were reviewed and approved by the ethics committee at the participating centres. Only patients who had given written informed consent to data collection prior to intervention were included.

The NEOS study was registered under Clinical Trails.gov as NCT04676672.

## RESULTS

In the NEOS, 100 patients with given consent were enrolled at 12 participating centres in 5 European countries and Hong Kong. One patient refused to attend the surgical procedure after providing written informed consent and, finally, 99 patients were treated with an E-vita OPEN NEO Stent Graft System. Clinical and morphological data of the 99 patients who underwent the intervention were collected prospectively. Major intraoperative parameters are listed in Table 1.

**Table 1:** Intraoperative procedure parameters

	*N* = 99
Total procedure time (min), mean (SD)	414.2 (98.6)
Cardiopulmonary bypass time (min), mean (SD)	233.2 (72.2)
Aortic cross-clamp time (min), mean (SD)	118.5 (47.6)
Visceral arrest time (min), mean (SD)	47.8 (21.6)
Cerebral perfusion time (min), mean (SD)	75.4 (44.6)
Primary cannulation site, *N* (%)	
Aortic arch	3 (3.0%)
Ascending aorta	19 (19.2%)
Axillary artery	60 (60.6%)
Brachiocephalic artery	8 (8.1%)
Femoral artery	6 (6.1%)
Left common carotid artery	3 (3.0%)
Core temperature of hypothermic circulatory arrest (rectal, bladder), *N* (%)	
<20°C	4 (4.0%)
20–25°C	31 (31.3%)
>25–30°C	64 (64.6%)
Cerebral perfusion, *N* (%)	
Antegrade	96 (97.0%)
Retrograde	3 (3.0%)
Cerebral perfusion technique, *N* (%)	
Unilateral	7 (7.1%)
Bilateral	63 (63.6%)
Trilateral	29 (29.3%)
Spinal cord fluid drainage, *N* (%)	
None	85 (85.9%)
Before intervention	3 (3.0%)
During intervention	3 (3.0%)
After intervention	10 (10.1%)
Distal diameter of E-vita OPEN NEO (mm), *N* (%)	
<24	16 (16.2%)
24–28	51 (51.6%)
30–36	45 (45.5%)
>36	2 (2.0%)
Length of stented portion (mm), *N* (%)	
120/130	83 (83.8%)
175/180	16 (16.2%)
Distal end of stented region, *N* (%)	
≤Th5	12 (13.0%)
Th6–Th7	56 (60.9%)
Th8–Th9	18 (19.6%)
≥Th10	6 (6.5%)

Data are presented as mean (SD) or as *n* (%), as stated above.

SD: standard deviation.

All patients were treated with the E-vita OPEN NEO Stent Graft System, with stent graft diameters available between 20 and 40 mm, and the stent graft length between 120 and 180 mm (Table 1). The distal suture zone (collar) was performed in 10 (10.1%) patients in zone 0, 13 (13.1%) patients in zone 1, 57 (57.6%) patients in zone 2 and 19 (19.2%) patients in zone 3. The branched configuration was used in 47 (47.5%) patients, straight configuration in 42 (42.4%) patients and the trifurcated configuration in 10 (10.1%) patients. The type of configuration used in relation to the arch zone distal anastomosis is detailed in Table 2.

**Table 2:** Configuration of E-vita OPEN NEO in relation to arch zone distal anastomosis

Distal suture zone (collar)	Configuration, *N* = 99		
	Trifurcated *N* = 10	Branched *N* = 47	Straight *N* = 42
Zone 0	7 (70%)	1 (2.1%)	2 (4.8%)
Zone 1	3 (30%)	4 (8.5%)	6 (14.3%)
Zone 2	0 (0.0%)	34 (72.3%)	23 (54.8%)
Zone 3	0 (0.0%)	8 (17.0%)	11 (26.2%)

Data are presented as *n* (%).

There were 66 men (66.7%) and 33 female (33.3%) with a mean age of 57.7 (standard deviation: 10.7) years (range, 31–76 years) (Table 3). Thirty-three (33.3%) were current smokers, and 27 (27.3%) were previous smokers. A total of 9 patients had connective tissue disease, of which Loeys–Dietz syndrome was recorded in 1 (1.0%) and Marfan syndrome in 8 (8.1%). The rate of patients who had previous sternotomy prior to the intervention with E-vita OPEN NEO Stent Graft System was 34 (34.3%). The other major comorbidities are documented in Table 3.

**Table 3:** Demographic data and medical history

	*N* = 99
Male	66 (66.7%)
Smoking	
Current	33 (33.3%)
Previous	27 (27.3%)
Connective tissue disease	
Loeys–Dietz syndrome	1 (1.0%)
Marfan syndrome	8 (8.1%)
Hyperlipidemia	32 (32.3%)
Arterial hypertension	84 (84.9%)
Renal insufficiency/renal failure	13 (13.1%)
Diabetes	6 (6.1%)
Surgery type	
Elective	59 (59.6%)
Emergent	23 (23.2%)
Urgent	17 (17.2%)
Number of interventions	
1	96 (97.0%)
2	2 (2.0%)
3	1 (1.0%)

Data are presented as *n* (%).

Patients underwent the following type of surgery: 59 (59.6%) were elective, 23 (23.2%) were emergent and 17 (17.2%) were urgent. Majority of the patients (*n* = 96, 97%) underwent only 1 intervention, while 2 (2.0%) patients had 2 interventions and 1 (1.0%) patient underwent 3 interventions (Table 3).

Of the 99 patients, 29 (29.3%) had aneurysm and 70 (70.7%) were diagnosed with dissection. Patients with dissection were further characterized as: 35 (35.4%) had acute type A aortic dissection, 2 (2.0%) had subacute aortic dissection and 33 (33.3%) had chronic type A aortic dissection. Of the patients with aneurysm, 15 (51.7%) were asymptomatic and 14 (48.3%) were symptomatic (Table 4). There were 2 (6.9%) patients who had aneurysm with contained rupture. Of the patients with dissection, the location of primary entry tear occurred in 27 (39.1%) patients in aortic arch, in 29 (42.0%) patients in ascending thoracic aorta and in 13 (18.8%) patients in descending thoracic aorta (Table 4).

**Table 4:** Morphology of aneurysm and dissections

Indication	*N* = 99
Acute (14 days) aortic dissection	35 (35.4%)
Subacute (15–90 days) aortic dissection	2 (2.0%)
Chronic (>90 days) aortic dissection	33 (33.3%)
Fusiform or saccular thoracic aortic aneurysm	29 (29.3 %)

Aneurysm	*N* = 29

Symptom status	
Asymptomatic	15 (51.7%)
Symptomatic	14 (48.3%)
Contained rupture	
No	27 (93.1%)
Yes	2 (6.9%)

Dissection	*N* ^a^ = 69

Location of primary entry tear	
Aortic arch	27 (39.1%)
Ascending thoracic aorta	29 (42.0%)
Descending thoracic aorta	13 (18.8%)

Data are presented as *n* (%).

a *N* denotes number of available patients at the time of database transfer. Data were assessed by the investigators.

### Mortality

Three patients (3.0%) died after the surgery and at discharge. The cause of death was visceral malperfusion (*n* = 1), multiorgan failure after aortic procedure (*n* = 1) and stroke (*n* = 1). The relatedness of death to the device and surgery was assessed by the investigators. None of the deaths were evaluated by the investigator as related to the E-vita OPEN NEO or to the TAA stent graft device used. Moreover, the death of the patient who had a stroke was not related to the E-vita OPEN NEO surgery. One patient was treated with E-vita OPEN NEO and extended with E-XL stent, and the visceral malperfusion of this patient was rated as probably related to the surgery performed using E-vita OPEN NEO. The other patient’s death with the multiorgan failure was rated as causal relation to the surgery.

Between discharge and 30 days after surgery, 2 additional patients died, and the cause of death was cardiovascular arrest (*n* = 1) and severe heart failure (*n* = 1). A possible relation of the death of the patient to the surgery was documented for the patient who died of cardiovascular arrest and for the patient with severe heart failure. Even though a relation to the E-vita OPEN NEO surgery cannot be excluded out for the patient with cardiovascular arrest, it is more likely that patient suffered from acute myocardial infarction as the patient had a coronary artery bypass graft surgery and coronary disease in the past.

**Figure 3:**
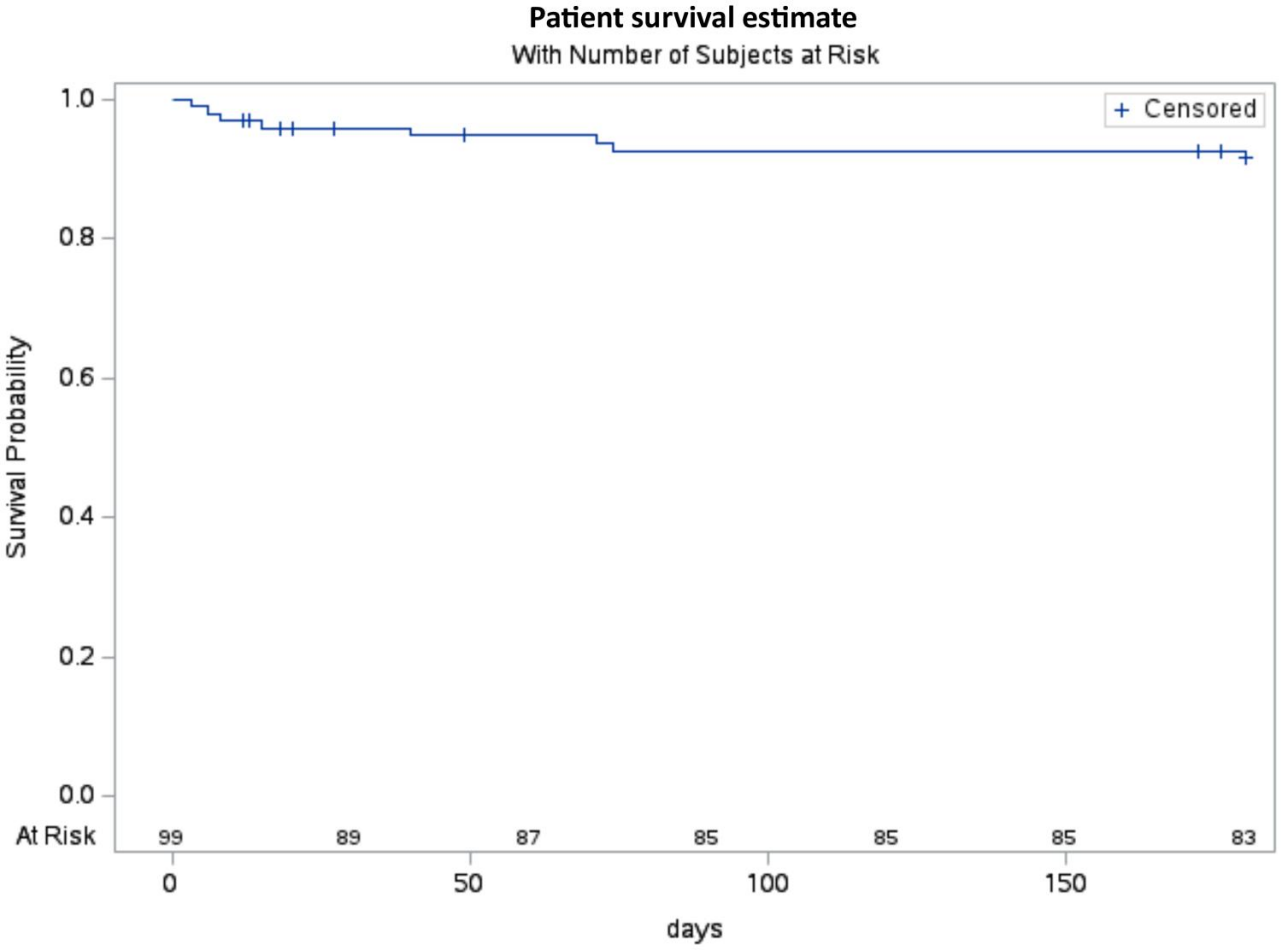
Kaplan–Meier survival estimate demonstrates a 6-month survival rate of 91.6%.

Between 30 days and at 3–6 months visit, death was reported for 3 additional patients. Of those, 1 patient died due to aortic-related mortality, which was evaluated to be probably related to the E-vita OPEN NEO surgery and possible related to the E-vita OPEN NEO. Later autopsy revealed periprosthetic infection. The remaining 2 patients died due to stroke (*n* = 1) and heart failure (*n* = 1). These 2 deaths were evaluated as not related to the E-vita OPEN NEO or to the E-vita OPEN NEO surgery. In total, there were 8 patients who died from the period of postintervention until 3–6 months follow-up visits. The estimated 6-month survival rate was 91.6% (standard deviation: 2.9) (Fig. 3).

### Device technical and treatment success

Successful delivery, deployment of the device in the planned location, patency of the device with the absence of device deformations and successful withdrawal were observed in 98 (99.0%) patients. Device technical success measured at 24 h postintervention was achieved in 96 (97.0%) patients. At discharge, treatment success was reported in 89.9% (*N* = 80/89). Treatment success at 3–6 months was achieved in 93.9% (*N* = 77/82). Furthermore, the rate of re-exploration due to bleeding within 24 h after final suture was necessary in 5 (5.1%) patients. Bleeding through graft material (non-stent graft region) after intervention to discharge was observed in 2 (2.2%) patients.

### Secondary end points

At discharge visit, 4.4% experienced new disabling stroke, 2.2% had new paraplegia, new paraparesis was recorded in 2.2% and renal failure requiring permanent (>90 days) dialysis or hemofiltration occurred in 3.3%. Between discharge and 3–6 months visit, no patients experienced new disabling stroke, new paraplegia or new paraparesis.

## DISCUSSION

This report presents the first interim results of the NEOS study until 3–6 months follow-up. In this study, the 30-day mortality rate is reported as 5.1% (*n* = 5), which compares favourably to the published literature on FET procedures. One of the latest systematic reviews and meta-analyses from 2022, which compared 85 FET studies and included almost 11 000 patients treated with different FET devices (not including the device in question), reported an average 30-day mortality of 8.0% [5]. Other meta-analyses on studies of FET procedures and devices calculate values ranging from 5% to 10% [6–9]. Diagnosis for 30-day or in-hospital mortality from several studies were mostly aorta-related death and included also visceral complications, cardiac failure, ischaemic stroke, pulmonary failure, intracerebral bleeding, abdominal aortic rupture and intestinal malperfusion [10–12]. At the time point from postintervention until 3–6 months, a total of 8 deaths were recorded (this includes the 5 deaths reported for 30-day mortality). Out of the 8 patients who died, 5 patients were indicated with chronic aortic dissection (*n* = 5/8), 2 patients indicated with thoracic aortic aneurysm (*n* = 2/8) and 1 patient indicated with acute aortic dissection (*n* = 1/8). In addition, there were 3 patients (*n* = 3/8) who had previous sternotomy. Since chronic aortic dissection and previous surgical operation (sternotomy) are associated with high postoperative risk, these factors might contribute to the death of the patients.

Although the treatment options for aortic arch pathology have broadened and improved substantially over the last decades, neurological complications remain a major concern in the management of extensive aortic arch pathologies [1]. New disabling stroke was reported for 4.4% of patients at discharge after the index procedure and no additional occurrences were found in the 3–6 months follow-up. This is favourable when compared with published literature, as systematic reviews and meta-analyses calculated values of 2.4% up to 13.7% with an average of about 6.9% for stroke rates after FET procedures [6, 13].

In the NEOS study, new paraplegia and new paraparesis occurrence was 2.2% each at discharge after the index procedure and no additional occurrence of each at 3–6 months follow-up. There was no relationship between the distal end of the stent graft in the descending aorta to the low incidence of paraplegia in these patients. Four systematic reviews and meta-analyses on FET studies were identified to calculate rates of paraplegia, ranging from 0.63% to 3.5% [5, 6, 8, 9]. None of these 4 systematic reviews and meta-analyses additionally reported values for paraparesis. Other systematic reviews and meta-analyses calculated values for spinal cord injury after FET procedures as 1.9% [14], up to 6.5% [7]. Smaller studies reported values for paraparesis ranging from 2.0% to 2.7% [15, 16], although including values reported for transient paraparesis. Hence, the NEOS study paraparesis and paraplegia rates are in line with published data in literature.

Renal failure is a potential complication of aortic dissection, resulting from renal hypoperfusion or infarction, secondary to the involvement of the renal arteries in the aortic dissection, or may be due to prolonged hypotension [2]. Acute kidney injury is reported to be 12.0% [5] and 15.5% [7] in systematic reviews and meta-analyses. Smaller clinical studies reported values of patients requiring permanent dialysis to be 1.4% up to 5.4% [10, 15–17]. These values are in line with the data obtained in the NEOS, as 3.3% of patients were reported to have renal failure requiring permanent (>90 days) dialysis or haemofiltration at discharge. It has to be noted that renal insufficiency and/or failure can be temporary but does not necessarily remain permanent.

Temperature management and optimal hypothermic level during circulatory arrest in the FET procedure have been a major topic of discussion among the cardiovascular surgeons over the past years. In the last years, there is a trend towards achieving warmer temperatures in the circulatory arrest, rather than the conventional deep hypothermia during aortic arch procedures as a means to reduce postoperative mortality and morbidity [18]. In complex aortic arch surgery, an optimal cerebral and organ protection strategy is key to achieve excellent results. For proximal aortic arch operations, which were associated with circulatory arrest times of about 30 min, retrograde cerebral perfusion in combination with deep hypothermia (<20°C) yielded similar results as antegrade cerebral perfusion in moderate hypothermia (20.1–28°C) [19]. However, FET implantation is associated with antegrade cerebral perfusion times of about 70 min. Consequently, the most effective protection technique is warranted to prevent neural injury. A meta-analysis including 5869 patients by Manoly *et al.* demonstrated a significantly reduced mortality rate and stroke rate in the moderate hypothermia plus antegrade cerebral perfusion group as compared to the deep hypothermia cohort [20]. In the NEOS study, the majority of patients were operated under moderate hypothermia (>25–30°C for 64.6% of patients) using antegrade perfusion (97.0%). This operative technique may further contribute to the excellent performance of the E-vita OPEN NEO Stent Graft System.

### Limitations

The present study is limited by the non-randomized setting and the lack of a control group.

## CONCLUSION

The low 30-day mortality rate, the comparable secondary end points to other FET studies and the high device technical success rate demonstrate excellent performance of the E-vita OPEN NEO Stent Graft System and its safe application when used within its indications for use. Long-term results are warranted to confirm the efficacy and durability.

## Data Availability

The data underlying this article were provided by JOTEC GmbH, a wholly owned subsidiary of Artivion, Inc. under licence/by permission. Data will be shared on request to the corresponding author with permission of JOTEC GmbH, a wholly owned subsidiary of Artivion, Inc.

## Notes

Presented at the EACTS Annual Meeting 2023, 4^th^ to 7^th^ October 2023, Vienna, Austria.

## ABBREVIATION

FET: Frozen elephant trunk
